# Hepatitis C: A Pharmacological Therapeutic Update

**DOI:** 10.3390/jcm10081568

**Published:** 2021-04-08

**Authors:** Sonia Santander Ballestín, David Gómez Martín, Sara Lorente Pérez, María José Luesma Bartolomé

**Affiliations:** 1 Department of Pharmacology, Physiology and Legal and Forensic Medicine, Faculty of Medicine, University of Zaragoza, 50009 Zaragoza, Spain; davidgm1512@gmail.com; 2 Department of Gastroenterology and Hepatology, Hospital Clinico Universitario Lozano Blesa, 50009 Zaragoza, Spain; slorentep@gmail.com; 3 Department of Human Anatomy and Histology, Faculty of Science, University of Zaragoza, 50009 Zaragoza, Spain; mjluesma@unizar.es

**Keywords:** hepatitis C, glecaprevir, pibrentasvir, sofosbuvir, velpatasvir, voxilaprevir, direct-acting antivirals

## Abstract

(1) Background: Hepatitis C is a high-prevalence disease, representing a global impact health problem. Lately, many changes have been made in treatment guidelines because of the commercialization of second-generation direct-acting antivirals due to their high effectiveness, few side effects and pangenotypic action. We address the pharmacological possibilities available and compare them with the current recommendations of the World Health Organization (WHO). (2) Methods: The search for articles was made through the PubMed database using different search strategies and we consulted technical data sheets of the treatments that have been included in the study. (3) Results: Combinations of “glecaprevir/pibrentasvir”, “sofosbuvir/velpatasvir” and “sofosbuvir/velpatasvir/voxilaprevir” have been recently incorporated. Phase II studies have shown that they are safe and effective therapies with very comfortable posologies and easy therapeutic adherence; furthermore, they suppose shorter treatment duration. Subsequently, phase III studies have shown they were effective for previously treated or compensated cirrhotic patients that previously had more complex treatment regimens. (4) Conclusions: These results suppose a simplification in Hepatitis C therapeutic approach, and open new study possibilities.

## 1. Introduction

Epidemiology: “Hepatitis C Virus” (HCV) is one of the leading causes of chronic liver disease associated with end-stage cirrhosis and hepatocellular carcinoma (HCC) [1,2,3]. Globally, it is estimated that approximately 71 million people live with chronic HCV infection. Fortunately, advances in treatment make it possible to cure more than 95% of infection cases, but the main limitation is access to diagnosis and treatment [1,2,3].

Approximately 90% of HCCs are associated with a known underlying etiology, most frequently chronic viral hepatitis (B and C), alcohol intake and aflatoxin exposure. In Africa and East Asia, the largest attributable fraction is caused by hepatitis B (60%), whereas in the Western world only 20% of cases can be attributed to “hepatitis B virus” (HBV) infection, while chronic hepatitis C appears to be the major risk factor [1,2,3,4].

Viral structure and replication: HCV is a small single-stranded, positive-strand RNA, enveloped virus, belonging to the *Hepacivirus* genus within the Flaviviridae family. Eight major HCV genotypes exist [5] and several subtypes have been identified; HCV genotypes show a characteristic distribution in different geographical regions. Genotypes 1, 2 and 3 exhibit a worldwide distribution. Genotypes 1 and 2 are endemic in West Africa while genotype 3 is endemic to the Indian subcontinent. Genotypes 4 and 5 are primarily found in Africa, and genotype 4 is particularly endemic in Egypt and Central Africa. Genotype 6 is endemic in Asia whereas the distribution of genotype 7 has not been fully assessed [6].

The virus particle is round or spherical in shape. The virus genome is located inside an icosahedral capsid, 9600 base pairs long and it encodes a unique polyprotein that is processed while being translated into at least 10 different proteins; three of which are structural (core, E1 and E2) and seven of which are non-structural (p7, NS2, NS3, NS4A, 51 NS4B, NS5A and NS5B); these proteins constitute pharmacological targets for different drugs available and under study [7].

Naturally, the virus exclusively infects humans. Its main target cells are hepatocytes, but it can infect other cell types such as B lymphocytes and dendritic cells [8]. HCV is usually non-cytopathic although HCV is cytopathic in the use of immunosuppressants [9]; the clinical manifestations it triggers are due to the host’s immune response [10].

HCV enters the cells by endocytosis after adhering to a membrane receptor. This is followed by an endosome fusion, leading to the release of the viral genomic charge into the cell. This genome acts as messenger RNA for the host cell translational machinery to synthesize the polyprotein, which is subsequently fragmented by proteases generating the different components of the virus. After polyprotein fragmentation, the new viral particles begin to assemble in the host’s sarcoplasmic reticulum and are subsequently released to the outside of the cell by exocytosis [7].

Viral transmission and risk factors: The ways of transmission of the virus are well known, although in most cases the route of transmission is not identified. Of the cases in which it is determined, sexual or parenteral routes are the most common. These have varied over time; for example, in the 20th century the fundamental mechanisms of transmission of the virus were the sharing of syringes in injecting drug users, blood transfusions or other derivatives and organ donation, the latter two prior to the establishment of viral RNA screening tests in blood [6,7].

Sexual transmission of the virus occurs through sexual contact without the use of barrier methods. The risk of HCV transmission by this route increases as the number of sexual partners increases [7].

Another mechanism of HVC transmission is occupational exposure, mainly in the field of health professionals. The incidence of seroconversion from a contaminated needle in the healthcare environment is estimated to be 1.8%. This risk is increased in developing countries where sterilization measures are often more deficient [7].

Cases of vertical transmission have been described, in which during the period of pregnancy the maternal viremia is increased and the cytotoxicity of the CD8 + T lymphocytes decreases, facilitating the perinatal transmission of the virus [6].

The highest transmission rates of the virus occur mainly in patients with acute cases of infection, which lead to the spread of more infectious variants [6]. On many occasions, it is difficult to determine the way of transmission of the virus in the patient affected by HCV since it presents a long incubation period that hinders its relationship with the source. In addition, there is a non-negligible percentage of acute HCV infections that are asymptomatic [6,7].

Clinical and natural history: HCV usually manifests itself in a small percentage of cases; close to 10% in the form of acute hepatitis, which does not usually present excessive severity and only 15% will present symptoms, although non-specific: nausea, vomiting, arthralgia, anorexia and choluria; jaundice being the most relevant and prevalent clinical sign in these patients. A high percentage of cases will evolve into a chronic form of HCV, but it is possible that spontaneous elimination may occur. Some of the factors that favor this process are the presence of the IL28B CC haplotype (rs12979860), a high CD4 + lymphocyte response or low levels of the INF-inducible protein [11].

The rest of the cases present a progression towards chronic liver disease, defined as the persistence of viral RNA more than 6 months after exposure to the virus, with the possibility of developing complications such as liver fibrosis that progresses to cirrhosis and development of hepatocarcinoma [1,2,3]. There are some factors that can modify the history of disease progression and accelerate the fibrotic process, some dependent on the virus (genotype 3, HIV or HBV coinfection), environmental factors (alcohol, tobacco) and factors inherent to the host (advanced age, male sex, IL28CC, hyperferritinemia, previous hepatic steatosis, insulin resistance) [12]. In certain cases, practically anecdotally, chronic liver disease can reverse spontaneously [13].

## 2. Materials and Methods

A review has been carried out in order to summarize the available results of experimental studies. To identify eligible papers, we performed a systematic literature search on the PubMED database and EASL guidelines. We used the following search terms: “Direct-acting antivirals or “Hepatitis C virus” or “Sofosbuvir” or “Velpatasvir” or “Voxilaprevir” or “Pibrentasvir” or “Glecaprevir”; using different combinations to find the articles and epidemiological studies that included the terms. We have excluded all the articles in which, in addition to HCV, other concomitant pathologies were involved, except for cirrhosis.

All articles were recovered and selected on the basis of presence/absence of the search criteria. To identify any articles that may have been missed during the literature search, reference lists of candidate articles have also been carefully checked.

The technical data sheets of the medicines that have been included were obtained through the Spanish Agency for Medicines and Health Products (AEMPS), and the European Medicines Agency (EMA).

## 3. Results

Currently, according to the guidelines of the World Health Organization (WHO), European guide (EASL) and guide of the Spanish Association for the Study of the Liver, the preferred treatments for HCV infection are “glecaprevir/pibrentasvir” or “sofosbuvir/velpastasvir”, since these drug combinations achieve cure rates higher than 95% [1,2,3].

### 3.1. Comparison of Pharmacological Possibilities Available

#### 3.1.1. Sofosbuvir

Sofosbuvir, an inhibitor of HCV NS5B RNA-dependent RNA polymerase, is a prodrug that undergoes intracellular metabolism and is transformed into uridine analog triphosphate, which is pharmacologically active and is incorporated into HCV RNA by NS5B polymerase and acts as a chain terminator [14]. This drug has been shown to be effective against different genotypes of virus 1b, 2a, 3a and 4a [15].

When its concentrations peak in plasma at 0.5–2 h after oral administration, more than half (60%) is bound to plasma proteins for transport. Administration together with food has no effect on the concentration peak in plasma or the area under the curve (AUC) [16].

Sofosbuvir metabolism has been seen at the liver level by four different enzymes that do not involve cytochrome P450, so there are no pharmacological interactions with inducers or inhibitors of the drug. The main metabolites obtained are the active triphosphorylated nucleoside (GS-461203) and, after dephosphorylation, the inactive metabolite GS-331007. At the liver level, it is transported by P glycoprotein (P-GP); hence, the plasma concentration of sofosbuvir will decrease with the presence of inducing compounds of this glycoprotein, such as rifampicin, carbamazepine, phenobarbital, rifabutin or phenytoin [1,16,17]. In patients with moderate or severe liver failure, exposure to the drug is increased, reaching 2.3 times higher levels [1,17].

Renal excretion is approximately 80% and is lower (15%), through the feces. Most of the compound found at the urine level is the metabolite derived from dephosphorylation, GS-331007 (78%), while only a small percentage of sofosbuvir is not metabolized (3.5%). The main route of elimination is through renal clearance, a fact to be considered for the administration of the drug in patients with impaired renal function (especially in filtrates below eGFR < 30mL/min/1.73 m^2^ or end-stage kidney disease undergoing hemodialysis) [1,17]. Nevertheless, there are some current studies that provide evidence on the safe use of sofosbuvir in patients with decreased glomerular filtration, and even on dialysis. However, clinical practice guidelines do not recommend its administration when glomerular filtration is <30 mL/min.

There are insufficient data regarding the use of the drug in human species. Animal studies do not show reproductive toxicity or effects on fetal development in rats or rabbits. However, it is preferable to avoid the use of sofosbuvir during pregnancy. Furthermore, in all treatment combinations that include ribavirin, extreme caution should be exercised and pregnancy should be avoided, as this drug has been shown to have teratogenic and embryocidal effects in all exposed species. If a male patient is taking this medication and his partner is of childbearing age, he should use a condom in cases of sexual intercourse to minimize the passage of ribavirin to the woman [18].

On the other hand, the combination of sofosbuvir with ledipasvir revealed, in addition to the common effects, an elevated number of enzymes such as creatine kinase, lipase or amylase, with no apparent clinical repercussion, this being a unique adverse effect of ledipasvir [19].

Due to pharmacological interactions in patients with concomitant treatment with Amiodarone, bradycardia and cardiac arrhythmias were reported, which could occur from hours after the start of treatment to two weeks later [18]. The mechanism involved is, to this day, unknown, although recent studies postulate that the cause could be due to interactions with the mechanisms of intracellular Ca^2+^ management [20].

In addition, cases of “Stevens Johnson syndrome”, drug skin reactions with eosinophilia and systemic symptoms (DRESS) and erythema multiforme have been reported [21].

#### 3.1.2. NS5A Inhibitors: Daclatasvir, Ledipasvir, Elbasvir, Pibrentasvir and Velpatasvir

Daclatasvir is an inhibitor of the non-structural protein NS5A identified by chemical engineering in 2010 [22,23,24]. Ledipasvir is also an NS5A inhibitor, with activity against HCV genotypes 1a, 1b, 4, 5 and 6 [22,23,24,25]. Elbasvir has activity mainly against genotypes 1 and 4 of the virus [26]. Pibrentasvir shows activity against the six major HCV genotypes [27]. Velpatasvir is a pangenotypic inhibitor; compared to the rest, this drug has a higher resistance barrier. These drugs act by the same mechanism of action, preventing the function of the NS5A protein and the formation of a protein complex necessary for the initiation of viral replication and influencing the subsequent assembly of the virion [27,28,29,30]. Pibrentasvir, Velpatasvir and their combinations will be addressed later.

There is no evidence showing variability in ledipasvir exposure in patients with severe liver failure, and even decompensated cirrhosis, compared to patients with normal liver function [1,17]. By itself, since renal excretion is minimal, its use in patients with renal failure would be safe, but since it is administered in combination with other direct-acting antivirals, mostly sofosbuvir, the limitations in this regard are determined by sofosbuvir. When combined with ribavirin, pregnancy is contraindicated due to the teratogenic effects. Administration in regimens that do not include ribavirin data are limited in human species, although no significant effects have been observed in rats and rabbits [23].

Elbasvir, meanwhile, is combined with other direct-acting antivirals, such as grazoprevir, which is an inhibitor of the viral protease NS3/4A. The limitations of this combination therapy are based on the second compound, since being a protease inhibitor it is contraindicated in the case of decompensated liver failure. Therefore, this treatment is recommended in naïve patients or patients previously treated with interferon regimens, without cirrhosis or with compensated cirrhosis (Child A) and for 1 and 4 viral genotypes [26]. According to the C-EDGE TN and C-EDGE TE studies, another recommendation is established, since differences were observed in the sustained virologic response (SVR) rate based on the patient’s baseline viremia, it being adequate when it was less than 800,000 IU/mL [31,32,33,34,35,36].

#### 3.1.3. NS3/4A Inhibitors: Glecaprevir and Voxilaprevir

NS3/4A cleaves the viral polyprotein at four sites, releasing proteins essential for viral maturation and infectivity. NS3/4A also impairs host-mediated viral elimination by cleaving host proteins, some of them related to the immune signal [37].

Glecaprevir is a pangenotypic inhibitor of NS3/4A. Voxilaprevir works by reversibly inhibiting the HCV NS3/4A protease with a pangenotypic action; compared to glecaprevir it presents a better resistance profile against genotype 1.

#### 3.1.4. New Drugs Included in the Strategic Approach Plan for HCV Treatment

The Strategic Approach Plan justifies the entry of new drugs for the treatment of Hepatitis C, including the new direct-acting antivirals since they are more effective, safer and better tolerated than predecessor therapies. The inclusion of these drugs is due to the simplification of therapy for patients, the reduction in their need for follow-up, the increase in infection cure rates in patients and the delay in the appearance of complications [38].

The drugs contemplated for treatment are MAVIRET^®^ (AbbVie Inc., North Chicago, IL, USA), which is a combination of two drugs “glecaprevir/pibrentasvir”; EPCLUSA^®^ (Gilead Sciences, Inc., Foster City, CA, USA), a combination of “sofosbuvir/velpatasvir”; and VOSEVI^®^ (Gilead Sciences, Inc., Foster City, CA, USA), resulting from the combination of three different active principles, sofosbuvir, velpatasvir and voxilaprevir, as rescue therapy.

##### Glecaprevir/Pibrentasvir

**Figure 1:**
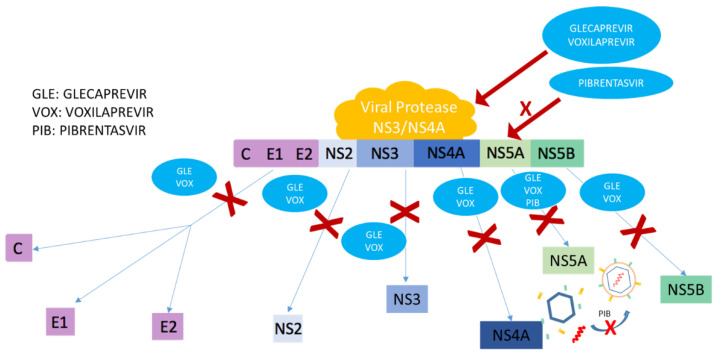
Mechanism of action of glecaprevir/voxilaprevir (viral protease inhibitors that prevent the cleavage of the Hepatitis C Virus (HCV) polyprotein and formation of replication active proteins) and pibrentasvir (inhibits NS5A, preventing replication of the virion and its subsequent assembly).

MAVIRET^®^ includes the combination of two of the HCV treatment pathways; Glecaprevir is a pangenotypic inhibitor of NS3/4A protease, which is essential to perform the proteolytic cleavage of the HCV polyprotein. Thus, this compound prevents the formation of active proteins essential for replication; Pibrentasvir, which is an inhibitor of the non-structural protein NS5A, hinders the formation of a protein complex necessary for viral replication and prevents subsequent assembly of the virion (Figure 1). This formulation has activity against the six principal HCV genotypes [25,39].

Both drugs reach a maximum concentration 5 h after oral administration, presenting an increase in the absorption of the active principles if administered with food compared to administration during fasting (glecaprevir (83–163%) and pibrentasvir (40–53%)). Likewise, the bioavailability of pibrentasvir triples when administered with glecaprevir compared to the administration of the drug alone. Both drugs bind strongly to plasma proteins, more so in the case of pibrentasvir, whose active principle is united at >99.9% [39].

The main route of elimination of both drugs is biliary excretion. They present a minimal renal clearance, mainly detected in glecaprevir, although it is less than 1% (0.7%) [35]. “Glecaprevir/pibrentasvir” has been studied in patients with different degrees of kidney failure with and without dialysis and a 56% increase in AUC and was found not to be clinically significant [1,17].

Exposure to the drug varies depending on the Child-Pugh stage, that is, on the patient’s prognosis of liver failure; hence, it is essential to evaluate this prior to the start of treatment. The active principle most affected by liver failure, like the rest of its pharmacological group, is the glecaprevir protease inhibitor. The administration of the combination of both principles is contraindicated in patients with Child-Pugh stages B and C; that is, in uncompensated cirrhotics [39].

Drugs present non-linear pharmacokinetics if the dose of pibrentasvir is higher than 120 mg, due to saturation of the efflux transporters [40]. Both active ingredients inhibit P-glycoprotein, breast cancer resistance protein and organic anion transport polypeptide. Due to the inhibition, interactions can occur with drugs that are substrates of the P-glycoprotein such as dabigatran, digoxin and etexilate, as well as with breast cancer resistance protein and organic anion transport polypeptide substrates (lipid lowering agents from the statin group). On the other hand, P-glycoprotein-inducing drugs can decrease plasma concentrations by reducing the therapeutic effect (rifampicin, carbamazepine, St. John’s Wort, phenobarbital, phenytoin) [1,17].

The most frequent adverse reactions detected are mostly mild, including headache, gastrointestinal disorders (diarrhea and nausea), fatigue and asthenia [25]. However, it is important to report suspected adverse reactions to update its data sheet, given its recent commercialization.

In two randomized open-label multicenter phase III studies (ENDURANCE I and III), patients infected by genotype 1 in an equal proportion were randomized to receive treatment with “glecaprevir/pibrentasvir” for 8 or 12 weeks; and on the other hand, patients with genotype 3 in a ratio of 2:1 to receive treatment with “glecaprevir/pibrentasvir” or with “sofosbuvir/daclatasvir” for 12 weeks. Subsequently, and in a non-randomized manner, a group of additional patients with genotype 3 were included to receive 8 weeks of treatment with “glecaprevir/pibrentasvir”. The comparison of “glecaprevir/pibrentasvir” treatment for 8 or 12 weeks, for patients infected with genotype 1, showed a sustained viral response rate at 12 weeks (SVR12). This was 99.1% (CI 95% 98–100) in the 8-week group; compared to 99.7% (95% CI 99–100) for the 12-week group. The results in the comparison between “glecaprevir/pibrentasvir” 12 weeks, versus “sofosbuvir/daclatasvir” 12 weeks showed a difference in the SVR12 rate of −1.2%; therefore, it was concluded that the treatment with “glecaprevir/pibrentasvir” vs. “sofosbuvir/daclatasvir” was not inferior. On the other hand, the group of genotype 3 patients treated for 8 weeks obtained an SVR12 rate of 95%, showing no inferiority when compared to the “glecaprevir/pibrentasvir” group for 12 weeks [41].

**Figure 2:**
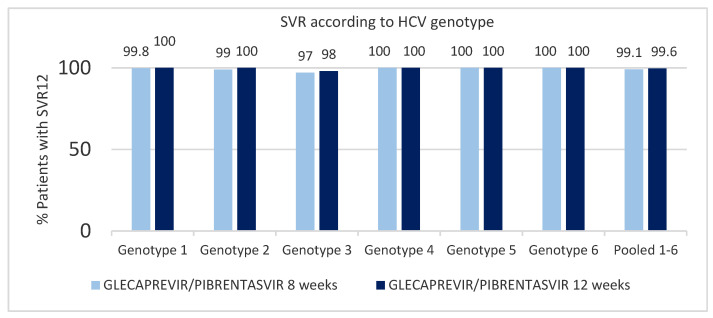
Percentage of patients with sustained viral response rate at 12 weeks (SVR12) according to genotype, comparison 8 vs. 12 weeks of “glecaprevir/pibrentasvir” treatment (modified from [42]).

The efficacy of this drug against other genotypes was tested in phase II or phase III clinical studies: EXPEDITION 2 and 4, ENDURANCE 1 to 4, SURVEYOR-I part two, SURVEYOR-II part one and two and SURVEYOR-II part four. These studies included different groups: patients with chronic HCV infections of all genotypes, patients with renal dysfunction and patients with HIV coinfection. None of the patients taking part in these studies presented cirrhosis. For patients with genotype 1 to 6 treated with a “glecaprevir/pibrentasvir” regimen for 8 weeks, the SVR12 rate was 98% (95% CI, 96.6–98.5%), compared to 99% (95% CI, 97.6–99.1%) in the group treated for 12 weeks, showing a non-significant difference in the SVR12 rate (*p* = 0.2). The relapse rates after treatment were 0.7% (95% CI 0.4–1.5%) and 0.3% (95% CI 0.1–0.8%) in the group of patients treated for 8 and 12 weeks, respectively. Excluding patients with non-virological failure, the rate of patients, modified by intention to treat, with SVR12 for both groups in all genotypes was on average 99.1% (Figure 2) [42].

In addition to the previously described studies, one phase II study, MAGELLAN-1, and two phase III studies, SURVEYOR-II part three and EXPEDITION-1, assessed the effectiveness of this drug combination for different genotypes and included patients pretreated with other direct-acting antivirals.

The study MAGELLAN-1 assessed the efficacy and safety of treatment with “glecaprevir/pibrentasvir” in patients infected with genotype 1 and previous treatment with another regimen of direct-acting antivirals being used at different doses and with or without Ribavirin. It consisted of three study groups:

- 1: Glecaprevir (200 mg) + Pibrentasvir (80 mg) during 12 weeks.
- 2: Glecaprevir (300 mg) + Pibrentasvir (120 mg) + Ribavirin, during 12 weeks.
- 3: Glecaprevir (300 mg) + Pibrentasvir (120 mg) during 12 weeks.

These groups were stratified according to the virus subtype and previous treatment received (which included an NS5A inhibitor, protease inhibitors without NS5A and others). The SVR12 rate, obtained by intention-to-treat analysis, in groups 2 and 3 was 92% (95% CI 81–97%); in group 1, which was treated with a lower dose, only six patients were included and all of them presented sustained virologic response [43].

In group 2, which included Ribavirin, sustained virologic response was achieved in 21/22 patients (95%, 95% CI 78–99) and among patients without Ribavirin 19/22 (86%, 95% CI 67–95%), with identical viral failure rates in both groups. Excluding patients who did not achieve sustained virologic response for non-virological reasons, the response rate was 100% in group 1 and 95% in the other two. With good safety profiles and the same reactions that we have previously discussed, the treatment was not interrupted in any case [43].

In SURVEYOR-II part three, the efficacy and safety of the treatment in patients with genotype 3 were assessed and included patients with compensated cirrhosis and/or who were previously treated against HCV. The study consisted of two treatment groups, one receiving treatment for 12 weeks and the other for 16 weeks. Naïve patients with cirrhosis treated for 12 weeks had an SVR12 of 98% (39/40 95% CI 87–99%); and those who had received previous treatment showed, after 16 weeks of glecaprevir/pibrentasvir treatment, an SVR12 of 96% (45/47 95% CI 86–99%). Patients without cirrhosis with previous treatment received 12-week regimens with a sustained virologic response rate of 91% (20/22 95% CI 72–97) and for 16-week regimens with a sustained virologic response rate of 95% (21/22; 95% CI 78–99); they presented a difference in SVR12 of −4.5% (CI 95% −23.6 to 13.9). It should be noted that within the patients with previous treatment, the group in which sofosbuvir was part of the treatment presented a sustained virologic response rate of 98% (41/42 CI 95% 88–99). Overall, the group of patients with cirrhosis showed a sustained virologic response rate of 97% (95% CI 90–99%) compared to patients without cirrhosis who had rates of sustained virologic response rate of 93% (95% CI 82–98%) [44].

The EXPEDITION-1 study included patients of genotypes 1, 2, 4, 5 and 6 with compensated cirrhosis, and, in addition, the studied group included patients without prior treatment and other patients treated with “ribavirin/pegylated interferon” or “sofosbuvir/ribavirin” with or without pegylated interferon with subsequent virological failure. “Glecaprevir/pibrentasvir” was administered in these patients for 12 weeks and SVR12 was assessed; in the intention-to-treat analysis, 99% was observed (95% CI 98–100), 69% of the patients presented adverse effects, majority being previously discussed, and the serious events that occurred had no direct relationship with the administration of the drug [45].

Finally, this study is complemented by EXPEDITION-8, which included patients with genotypes 1–6, naïve and with compensated cirrhosis who received treatment for 8 weeks and compared SVR12 with the pre-established cut-off point based on the studies of treatment for 12 weeks. In this study, sustained virologic response rates of 99.7% were achieved in the protocol analysis and 97.7% in the intention-to-treat analysis, thus demonstrating that the treatment regimen is valid for this group of patients. This strengthens the first 8-week pangenotypic treatment for HCV [46].

##### Sofosbuvir/Velpatasvir (EPCLUSA^®^)

The dual therapy, called EPCLUSA^®^, is a combination of the previously discussed sofosbuvir, a pangenotypic inhibitor of NS5B polymerase; and velpatasvir, a pangenotypic inhibitor that acts on the non-structural protein NS5A, necessary for various processes of the virus life cycle, including replication, virion assembly and complex interactions with host cell functions. Compared to the rest of the NS5A inhibitors, this drug has a higher resistance barrier [41]. Efficacy as a pangenotypic therapy was assessed in four phase III studies as shown in Table 1.

##### Sofosbuvir/Velpatasvir/Voxilaprevir (VOSEVI^®^)

Another drug included in the Strategic Approach Plan, VOSEVI^®^, is a combination of three different active ingredients, each of which acts on a different pharmacodynamic target and at different points in the viral replication cycle.

This drug, in addition to sofosbuvir and velpatasvir, includes voxilaprevir, which works by reversibly inhibiting the HCV NS3/4A protease, so that the fragmentation of the viral polyprotein is prevented, hence avoiding the formation of the rest of the components (Figure 1). One of the peculiarities of this drug compared to the rest of the protease inhibitors is that it presents a better resistance profile against genotype 1. Like the rest of the active principles of the drug, it has pangenotypic activity [47,48,49,50,51].

The administration of the drug is performed orally in the form of a single tablet containing the three principles; there is no modification of the pharmacokinetics of sofosbuvir with respect to that previously explained [45]. Velpastasvir and voxilaprevir reach a maximum plasma concentration four hours after administration. When it is administered together with food, there is an increase in Cmax and AUC. Both active ingredients (velpastasvir and voxilaprevir) have a high plasma protein binding, greater than 99% [52].

The route of elimination of sofosbuvir is renal, in the form of an inactive metabolite, as already mentioned, while the other two active principles are eliminated through the bile and are subsequently excreted in the feces; elimination of velpastasvir is mainly unchanged (77%), and in lower amounts as metabolites are generated at the liver level by CYP2B6, CYP2C8 and CYP3A4. Voxilaprevir is eliminated unchanged (40%) and the rest in the form of metabolites generated in part at the intestinal level and the rest as a result of its hepatic metabolism through CYP3A4 [47].

There are no modifications in patients with mild or moderate renal insufficiency, although in patients with severe renal insufficiency or terminal disease, as with sofosbuvir, its use is not recommended due to the lack of studies that support it. For liver failure, it does not need any type of adjustment in Child A stage (mild); however, it is not recommended in the other stages due to the presence of a protease inhibitor voxilaprevir that when administered in class B or C patients, undergoes a 500% increase in exposure [53].

The adverse reactions identified with administration of this drug are mild, the most frequent being headache. It should be noted that due to the inhibition of organic anion transport polypeptide 1B1 and organic anion transport polypeptide 1B3 by voxilaprevir, increases in bilirubin can be observed. This increase is less than 1.5 times the upper limit of normality and normalizes at the end of treatment, presenting no clinical relevance [52,53,54].

Since sofosbuvir is one of the components, it will present the same adverse reactions that have been previously exposed. The drug has recently been approved and there may be adverse reactions not detected in the pre-marketing studies that must be reported in order to update its technical data sheet.

**Figure 3:**
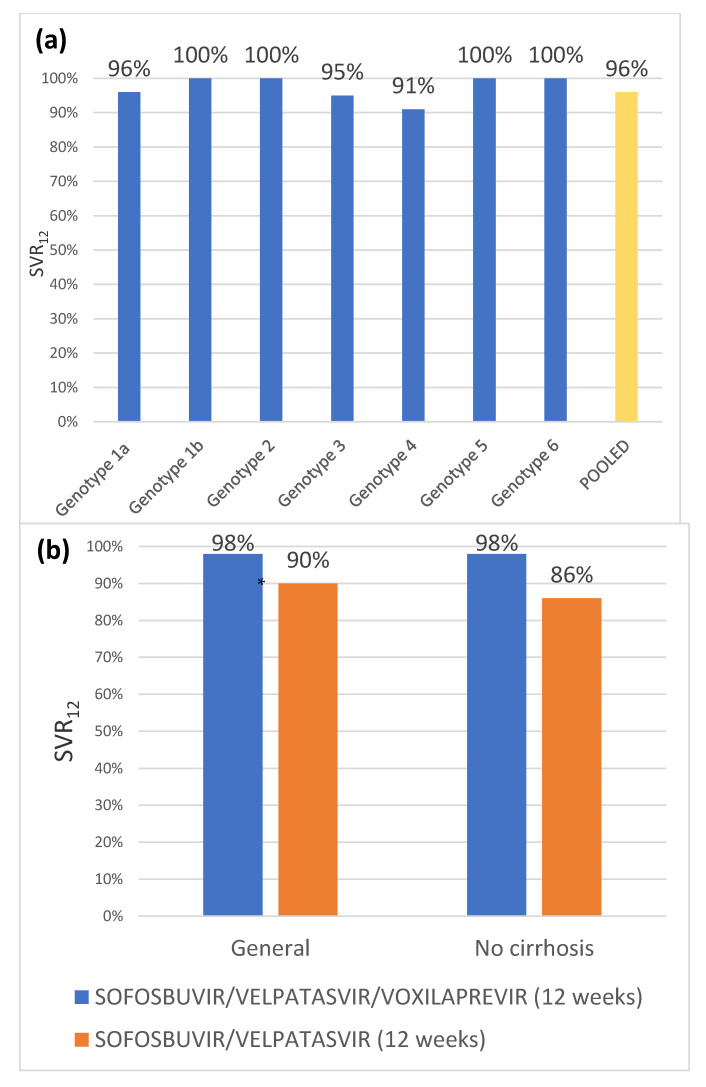
(**a**) Phase III studies POLARIS-1; (**b**) Phase III studies POLARIS-4. * The result of 98% was higher than the established objective of 85%.

Three phase II studies were carried out, the conclusions of which are set out in Table 2 [55,56,57]. In addition to these phase II studies, some phase III studies have been conducted with larger populations (Table 3):

In the POLARIS-1 and POLARIS-4 studies (Figure 3), patients had been previously treated with other direct-acting antivirals. In the first case, patients previously received NS5A inhibitors; and in the second, they received treatment with a different direct-acting antiviral. Both studies included patients of the 6 main genotypes and patients with and without cirrhosis (excluding those with decompensated liver disease).

The study POLARIS-4 consists of two groups of patients: the first receiving “sofosbuvir/velpatasvir/voxilaprevir” treatment, and the second receiving “sofosbuvir/velpatasvir”, both for 12 weeks. In this study, patients were randomized 1:1 for genotypes 1–3 and patients with genotypes 4–6 were included in the first group. They were stratified by genotype and presence or absence of cirrhosis. The SVR12 rate for the first group was 98% (95% CI 95–99%), significantly higher than the pre-established objective of 85% (*p* < 0.001), whereas the second group experienced an SVR12 rate of 90% (95% CI 84–94%). It should be noted that, among patients with cirrhosis, the sustained virologic response rate was 98% in the first group and 86% in the second [58].

Naïve patients were studied in POLARIS-2 and POLARIS-3, the first of which included patients of all genotypes with and without cirrhosis, except for genotype 3 patients with cirrhosis, who were included in POLARIS-3.

POLARIS-2 consisted of two study groups of patients, the first of which received “sofosbuvir/velpatasvir/voxilaprevir” for 8 weeks, while the second group received “sofosbuvir/velpatasvir” for 12 weeks. Patients with genotypes 1, 2, 3 or 4 were randomized in a 1:1 ratio and those with genotype 5 and 6 were assigned to the first group. Sustained virologic response rates in the first group were 95% (95% CI 93–97%) and 98% (95% CI 96–99%) for the second group. Given that the difference in 95% CI between the sustained virologic response rates was −6.2%, which was below the pre-established value of −5%, it was not possible to establish the non-inferiority of 8 weeks of treatment with “sofosbuvir/velpatasvir/voxilaprevir” [58].

The POLARIS-3 study, from patients with genotype 3 and cirrhosis, consisted of two treatment groups. The first group was treated with “sofosbuvir/velpatasvir” for 12 weeks, and the second was treated with “sofosbuvir/velpatasvir/voxilaprevir” for 8 weeks. The sustained virologic response rate in both groups was 96% (95% CI 91–99%), being significantly higher than the preset goal of 83% (*p* < 0.001). Two patients in the “sofosbuvir/velpatasvir/voxilaprevir” group and one in the “sofosbuvir/velpatasvir” group relapsed at week four post-treatment. Furthermore, in the first group a patient presented virological failure during treatment [58].

## 4. Discussion

Advances in the pharmaceutical industry are essential to establish improvements in the treatment regimens of patients infected with HCV, either to improve safety profiles, decrease drug interactions, facilitate administration and dosage or decrease treatment time. The new drugs included in this review are proposed as new therapies and safe alternatives for the treatment of HCV infection.

Regarding “glecaprevir/pibrentasvir”, the ENDURANCE 1 and 3 studies [41] showed good results for the treatment of two of the most prevalent genotypes, 1 and 3, with virological response rates of >99% for genotype 1 and of 95% for genotype 3. In addition, these treatments demonstrated a high level of safety, with low adverse reactions, and being the treatments reported in most mild cases, this represents a great advantage both for the patient’s health and for reducing the lack of adherence to treatment [41].

It should be noted that for the treatment of genotype 1, the administration for 8 or 12 weeks was compared and showed no inferiority, which implies a substantial reduction in the duration of the treatment, this being favorable for patients, and a minimization of costs for the patient. Besides the national health system, others have addressed the possibility of decreasing the treatment to 8 weeks; the ENDURANCE-4, SURVEYOR-II and ENDURANCE-2 studies [42], which examined genotypes 2, 4, 5 and 6. The comparison of different treatment times showed an virological response rates of 98% for the 8-week treatment, and 99% for the 12-week treatment. Additionally, both cases had comparable subsequent relapse rates. Altogether, this implies non-inferiority of the 8-week treatment. These studies open ways to reductions in the treatment period in the most favorable cases (naïve patients, without cirrhosis), compared to previous therapies in which this had only been possible in patients with genotype 1 and without previous treatment with “sofosbuvir/ledipasvir” [42]. This fact was demonstrated as regards pangenotypic treatment in EXPEDITION-8 [46]; when comparing the SVR in the 8-week treatment against a cut-off point based on the studies with 12 weeks, it was demonstrated in the statistical analysis that it was positioned as a pangenotypic therapy in a naïve patient, with or without compensatory cirrhosis [43].

Furthermore, this combination has been shown in several studies to be a valid option for a group of patients who until now had very limited options, those who had previously received treatment with direct-acting antivirals and had not had an adequate viral response. MAGELLAN-1’s study [43] of the effectiveness against different resistances to previous regimens with similar sustained virologic response rates demonstrated the association or not of ribavirin to treatment, concluding that this did not increase the efficacy of the treatment, nor was it necessary. In SURVEYOR-II part three [44] in patients with genotype 3 (cirrhosis and/or previous treatment) it was observed that the regimen was effective and well tolerated in this type of patient, and high rates of virological response were obtained in prolonged treatments for 12 weeks or, in patients with pretreatment, including sofosbuvir, 16 weeks. EXPEDITION-1 [37], for patients of all genotypes, excluding the genotype 3 that is addressed in SURVEYOR-II, and who had received treatment with “ribavirin/pegylated interferon” or “sofosbuvir/ribavirin” with or without pegylated interferon showed virological response rates close to 100% with 12-week treatments, regardless of whether they had been previously treated with other direct-acting antivirals [43,44,45].

All of the above mentioned would simplify most treatments. They can be administered in any viral genotype and in patients with compensated cirrhosis or in patients who previously received other direct-acting antivirals treatment in shorter regimens than those present so far: 8 weeks in the most favorable cases, 12 weeks for previous treatments or cirrhosis and 16 weeks in the specific case of genotype 3 with previous other direct-acting antivirals treatment.

The main problem when taking this combination of active principles is the dosage. Currently, the recommended dose is “glecaprevir (300 mg daily)/pibrentasvir (120 mg daily)”, and the presentation of the drug contains a dose of “glecaprevir (100 mg daily)/pibrentasvir (40mg daily)”. Therefore, three tablets must be administered simultaneously once a day orally.

The second drug examined in this review is “sofosbuvir/velpatasvir”, which could be administered in patients with compensated cirrhosis or without cirrhosis for 12 weeks as usual therapies [46,47,48]. On the other hand, it constitutes a first-line treatment option in patients with decompensated cirrhosis (Child B), since they lack a protease inhibitor that can be administered. The regimen that obtained the best virological response rates in this situation was the administration of “sofosbuvir/velpatasvir/ribavirin” for 12 weeks [49].

The triple therapy “sofosbuvir/velpatasvir/voxilaprevir” interacts with all the pharmacological targets of HCV existing up to the moment and acts at different points in the viral cycle. In addition, the dosage is a single tablet per day containing “sofosbuvir (400 mg daily)/velpatasvir (100 mg daily)/voxilaprevir (100 mg daily)”.

Various phase II studies assessed its efficacy in patients previously treated with other direct-acting antivirals. In one of them, Lawitz et al. [54] assessed whether or not it was effective to add ribavirin in the treatment for this type of patient to achieve better results and increased virological response rates. All the patients included in the study (*n* = 49) were infected with genotype 1. The study results showed that the cure rate in these patients was 100% without differences between both groups (with or without ribavirin), thus obviating the need for ribavirin in this subgroup of patients. So far, ribavirin uptake had been considered mandatory in many circumstances in which a poor response to treatment was anticipated, such as cirrhosis. Besides, therapeutic management of this compound is complex due to the need for dose adjustment depending on the patient’s weight and dosage, which does not facilitate its administration only once a day. Therefore, removal of ribavirin simplifies the treatment regimen and makes it easier for patients to adhere to it.

Furthermore, Lawitz et al. [55], in a different study with a larger sample (*n* = 197), showed that it was impossible to reduce the treatment regimen below 8 weeks, even in the most favorable patient groups (without cirrhosis or previous treatment), since the virological response rates only reached 71% of the patients if they were treated for 6 weeks. However, they observed that the treatment for naïve patients was effective for 8 weeks, including patients who had cirrhosis; and that for previously treated patients the appropriate regimen to obtain an virological response rates was 12 weeks. Gane et al. [56] studied this fact for the rest of the genotypes, showing the same results: all genotypes presented high virological response rates after 8-week treatment in case of naïve patients, and 12-week treatment for those previously treated [54,55,56].

The results obtained in these studies were used to formulate hypotheses which were then contrasted in phase III studies with larger population samples.

POLARIS-1 and 4 [55] showed that prolonged treatment for 12 weeks, the same as the usual therapies, obtained high rates of virological response rates close to 100% in all types of patients: with or without cirrhosis, different genotypes of the virus, with or without prior treatment (including polymerase A inhibitors). The virological failure mechanisms that occurred in the previous treatments were studied, and even in patients whose treatments were based on modifications in the viral genome, the response rate was adequate without affecting the overall response. This shows greater potency of the drug, since, even with viral resistance mechanisms, it does not significantly decrease the virological response rate of the drug [57].

In addition, the rate of adverse effects was similar in the treated and placebo groups and was mild in nature (POLARIS-1). The presence of adverse effects had previously been a reason for discontinuation of treatment [57], but was not the case in this study.

However, the sample is not significant in certain cases and it is not appropriate to extrapolate the results to the overall set of patients, especially for genotypes 2, 5 and 6.

Finally, POLARIS-3 [58] analyzed the only possibility not studied, due to the exclusion in the previous study: patients with genotype 3 and cirrhosis, just as the previous study compared the two treatment guidelines. In this case, it was possible to demonstrate the non-inferiority of the studies; both obtained the same virological response rate. It is worth noting the increase in adverse reactions in the group receiving “sofosbuvir/velpatasvir/voxilaprevir” compared to “sofosbuvir/velpatasvir”, these being entirely mild. The more frequent adverse reaction was the gastrointestinal type if voxilaprevir was associated, and not influencing the continuity of the regimen of treatment [58].

Compared with treatment with “glecaprevir/pibrentasvir”, this treatment has shown that not only in the most ideal situations without cirrhosis and without prior treatment is it possible to decrease the duration of therapies; the results of POLARIS-3 [58] open ways to develop new studies that allow evidence-based 8-week treatments for patients with different genotypes and with cirrhosis.

## 5. Conclusions

Both phase II and phase III studies report that the “glecaprevir/pibrentasvir”, “sofosbuvir/velpatasvir” and “sofosbuvir/velpatasvir/voxilaprevir” combinations all show good safety and efficacy profiles. Currently, cohort studies from real clinical practice have already been published showing an efficacy and safety profile of these combinations similar to those of the registry studies.

The “glecaprevir/pibrentasvir” combination has been shown to be a pangenotypic therapy that reduces the treatment time to 8 weeks in a specific group of patients; those who have not received previous treatment, any genotype and with compensated cirrhosis. There are ribavirin-free guidelines for the treatment of chronic hepatitis C virus in patients with cirrhosis.

These treatments are imposed as safe and effective for a group of patients previously devoid of therapeutic options, those who had received a previous regimen with other direct-acting antivirals and who had post-treatment failure or relapse.

The combination of “sofosbuvir/velpatasvir” has been positioned as the only pangenotypic treatment available for patients with decompensated cirrhosis. In the same way as their effect in previously treated patients, they involve drugs with high resistance barriers against the virus. Thus, due to its safety and potent action it constitutes the first line of treatment in current guidelines.

Currently, the most frequent cause of treatment failure is lack of adherence. Since the drugs examined here represent safe, well-tolerated regimens, with low adverse reactions and shorter duration of treatment than their predecessors, they should be considered as the first option in groups of patients with a history of poor adherence to other therapies.

Triple therapy “sofosbuvir/velpatasvir/voxilaprevir” should be evaluated for each virus genotype; the efficacy of 8-week treatment regimens, since, for genotype 3, it has been shown to be effective, including for patients with cirrhosis or previous treatments with other direct-acting antivirals; since, it could mean a decrease in cost and an improvement in treatment adherence; meanwhile, the duration decreases considerably.

Consideration should be given to evaluating the option of carrying out a study with a larger sample of genotype 2, 4, 5 and 6 patients, to affirm that “glecaprevir/pibrentasvir” constitutes a 12-week treatment regimen suitable for previously treated patients, because the current studies have insufficient samples to extrapolate the results to the general population.

## Figures and Tables

**Table 1:** ASTRAL studies: Efficacy as a pangenotypic therapy in phase III studies (TN: Treatment Naïve; TE: Treatment Experienced; NC: Non-Cirrhotic; CC: Compensated Cirrhosis; CTP-B Cirrhosis: Child-Turcotte-Pugh B Cirrhosis).

	Genotypes	Results
ASTRAL 1	GENOTYPES 1, 2, 4–6 TN, TE NC, CC	Compared to placebo, SVR_12_ was obtained >97% [47]
ASTRAL 2	GENOTYPE 2 TN, TE NC, CC	Compared against “sofosbuvir/ribavirin”, it showed statistical superiority (difference 5.2% CI95 (0.2–10.3%) and SVR 99% [48]
ASTRAL 3	GENOTYPE 3 TN, TE NC, CC	Compared against “sofosbuvir/ribavirin”, it showed statistical superiority (difference 14.8% CI95 (9.6–20%) and SVR rates 95% [48]
ASTRAL 4	GENOTYPES 1–4 and 6 TN, TE CTP-B Cirrhosis	“sofosbuvir/velpatasvir” 12 weeks vs. “sofosbuvir /velpatasvir/ribavirin” 12 weeks and “sofosbuvir /velpatasvir”24 weeks. The best results were obtained in “sofosbuvir /velpatasvir/ribavirin” with SVR 94% [49]
ASTRAL 5	GENOTYPES 1–4 TN, TE NC, CC	“sofosbuvir /velpatasvir” 12-weeks efficacy and safety in HCV patients coinfected with HIV-1 [50]

**Table 2:** Phase II studies of triple therapy. ^1^DAA (Direct action antivirals); ^2^SOF (Sofosbuvir); ^3^VEL (Velpatasvir); ^4^VOX (Voxilaprevir); ^5^RBV (Ribavarin).

	Lawitz [46]	Lawitz [47]	Gane [48]
Genotype			2
	1	1	3–4
			6
Previous treatment with ^1^DAA	Yes	Yes	Yes
Patients with cirrhosis	Yes	Yes	Yes
Treatment groups	^2^SOF/^3^VEL/^4^VOX + ^5^RBV ^2^SOF/^3^VEL/^4^VOX	Non previous treatment No cirrhosis (6 weeks) Cirrhosis (8 weeks) No cirrhosis (8 weeks) Cirrhosis + RBV (8 weeks) Previous treatment Cirrhosis (12 weeks) No cirrhosis (12 weeks)	No previous treatment No cirrhosis (6 weeks) Cirrhosis (8 weeks) Previous treatment No cirrhosis (12 weeks) Cirrhosis (12 weeks)

**Table 3:** POLARIS studies: Phase III studies of triple therapy. ^1^DAA (Direct action antivirals); ^2^SOF (Sofosbuvir); ^3^VEL (Velpatasvir); ^4^VOX (Voxilaprevir); * Genotype 3 + Cirrosis excluded.

	Polaris 1	Polaris 2	Polaris 3	Polaris 4
Previous treatment	NS5A inhibitors	-	-	^1^DAA except NS5A inhib
Genotype	1–6	1–3 * 4–6	3	1–6
Comparison group	Placebo	^2^SOF/^3^VEL 12 weeks	^2^SOF/^3^VEL 12 weeks	^2^SOF/^3^VEL 12 weeks
Group to assess	^2^SOF/^3^VEL/^4^VOX 12 weeks	^2^SOF/^3^VEL/^4^VOX 8 weeks	^2^SOF/^3^VEL/^4^VOX 8 weeks	^2^SOF/^3^VEL/^4^VOX 12 weeks
